# Are sleep disturbances involved in suicidal risk? Current insight and therapeutical considerations

**DOI:** 10.1192/j.eurpsy.2026.10387

**Published:** 2026-07-31

**Authors:** W. McCall

**Affiliations:** Psychiatry and Health Behavior, Medical College of Georgia, Augusta, United States

## Abstract

**Abstract:**

Psychiatrists have limited ability to predict near-term risk of suicidal behavior, and the positive predictive value of algorithms is not much better than chance. Insomnia is now a recognized risk factor for suicidal phenomena in persons with mood disorders or psychotic disorders. Hyperarousal is one explanation for the link between insomnia and suicide risk, and laboratory evidence of physiological hyperarousal can be shown with assays of the autonomic nervous system, including measurement of the pupillary light reflex or heart rate variability. We hypothesize that elevated levels of physiological arousal impair executive decision making and put a person at risk of suicidal behavior. These observations lead to the premise that treating insomnia may reduce suicidal ideation or behavior. Published studies show that improving insomnia in people with mood disorders or psychotic disorders is associated with reduced suicidal ideation. Both psychotherapy and medications that improve insomnia may have a role in reducing suicidal ideation. More research is needed to examine the impact of reducing insomnia on suicidal behavior.

**Disclosure of Interest:**

W. McCall Grant / Research support from: Axon Medical Technologies, Consultant of: Axon Medical Technologies; Haleon; Carelon; Sigma Stim, Speakers bureau of: Idorsia.

